# The relationship between physical activity and career decision-making self-efficacy in Chinese college students: the mediating roles of self-control and social anxiety

**DOI:** 10.3389/fpsyg.2025.1541211

**Published:** 2025-01-29

**Authors:** Yuan Fang, Tingting Xu, Maosheng Ye, Changquan Li

**Affiliations:** ^1^School of Physical Education, Qiannan Normal University for Nationalities, Duyun, Guizhou, China; ^2^Library, Qiannan Normal University for Nationalities, Duyun, Guizhou, China; ^3^Department of Physical Education and Research, China University of Mining and Technology-Beijing, Beijing, China

**Keywords:** physical activity, career decision-making self-efficacy, self-control, social anxiety, mediating

## Abstract

**Objectives:**

Enhancing career decision-making self-efficacy is an effective approach to improving university graduates' employment quality and speed. This study aims to explore the relationships among physical activity, career decision-making self-efficacy, self-control, and social anxiety to provide new perspectives and directions for enhancing university students' career decision-making self-efficacy.

**Methods:**

Within the framework of this research endeavor, a cohort of 1,955 university students (*N* = 1,955) from 14 universities distributed throughout China was surveyed. The initial data was entered and stored by means of Microsoft Excel. Subsequently, SPSS version 26.0 was employed to execute a comprehensive set of statistical analyses on the data, including descriptive statistics, a normality test, a reliability test, an exploratory factor analysis (aimed at addressing common method bias), a correlation test, and a regression analysis. In the present study, a structural equation model was formulated via the utilization of AMOS 24.0 software, and the Bootstrap approach was implemented. A total of 5,000 samples were randomly drawn for the purpose of validating the research hypotheses, with the determination being based on whether the 95% confidence interval encompassed the value of 0.

**Results:**

(1) Physical activity was found to positively predict career decision-making self-efficacy, as evidenced by (Estimate = 0.590, *p* < 0.001). (2) Self-control was demonstrated to play a mediating role in the connection between physical activity and career decision-making self-efficacy, with the effect indicated by (Estimate = 0.075, 95%CI(0.042, 0.113)]. (3) Social anxiety was likewise shown to assume a mediating role within the relationship between physical activity and career decision-making self-efficacy, as manifested by [Estimate = 0.009, 95%CI(0.002, 0.020)]. (4) A chain mediating effect was observed between physical activity and career decision-making self-efficacy for the combination of self-control and social anxiety, with [Estimate = 0.032, 95%CI(0.008, 0.057)].

**Conclusion:**

These findings provided significant theoretical support for physical activity as an effective means to enhance university students' career decision-making self-efficacy and offered references for designing sports activity programs. Furthermore, this study offered new perspectives and directions for understanding and enhancing university students' career decision-making self-efficacy.

## 1 Introduction

The employment of college students upon graduation has consistently been a matter of utmost concern within Chinese society (Liu, 2021). In grappling with this issue, the core elements influencing the employment problem of college students can be classified into two main aspects. The external aspect chiefly pertains to the employment environment and employment services, whereas the internal factors encompass college students' employment concept and career decision-making self-efficacy (Xu and Tian, 2024; Zhang et al., 2022).

Career decision-making self-efficacy was developed based on the Theory of Self-Efficacy (Bandura, 1977) and the Theory of Career Maturity (Crites, 1961). Career decision-making self-efficacy is defined as “an individual's degree of belief that he or she can successfully complete tasks necessary for making career decisions” (Betz and Luzzo, 1996). It exhibits a close correlation with the scope of career choices available to college students and their motivation in career selection behaviors. Augmenting career decision-making self-efficacy represents an efficacious approach to enhancing the quality and expediting the process of college students' employment (Shen et al., 2009).

Given the crucial significance of career decision-making self-efficacy for college students' employment, extant studies have revealed that internship experience, work experience, and career choice status have an impact on college students' career decision-making self-efficacy (Chen et al., 2022; Plakhotnik et al., 2020). Komarraju et al. (2014), drawing on Bandura's (1977) Social Cognitive Framework, devised a career decision-making self-efficacy enhancement program that also exerted an influence on the students. These investigations primarily concentrated on practical experiences and the implementation of career courses to boost career decision-making self-efficacy.

Nevertheless, sports, being a crucial element of university students' campus life, offer a novel approach to enhancing career decision-making self-efficacy. In this study, physical activity is defined as an individual's active participation and sustained commitment in sports-related pursuits, which involves proactively arranging sports time, routinely engaging in a wide range of sports activities and competitions, and actively striving to improve sports skills. Previous research has established the impact of physical activity on self-efficacy (Reverdito et al., 2017). According to the self-efficacy theory (Bandura, 1977), individuals develop self-efficacy via four main channels. In the domain of physical activity, it provides individuals with opportunities to acquire such experiences. More precisely, during sports competitions, individuals encounter mastery experiences that shape their perception of their capabilities and are associated with career decision-making self-efficacy. Observing sports idols or teammates provides vicarious experiences, thereby enhancing confidence. Social support within the sports context serves as a form of social persuasion, strengthening self-belief. Additionally, the modulation of emotional and physiological states helps manage stress and anxiety related to career decision-making. Moreover, relevant studies have also verified the positive effect of physical activity on career decision-making levels and career maturity (Kim, 2018, 2019; Fang, 2022).

Based on the potential association between physical activity and career decision-making self-efficacy, as well as the confirmation by existing studies of the correlation between physical activity and college students' career decision-making and development, we posit Research Hypothesis 1 (H1): physical activity has a significant positive predictive effect on college students' career decision-making self-efficacy.

Self-control is defined as an individual's capacity to actively modify their dominant responses and regulate their own behaviors, thoughts, and emotions, an ability to remain focused in order to achieve long-term goals (Englert et al., 2020; Hagger et al., 2010). Related studies have demonstrated a bidirectional relationship between exercise and self-control. Specifically, individuals with greater self-control display higher exercise performance and persistence levels, and long-term adherence to physical exercise, in turn, augments self-control (Boat and Cooper, 2019). Furthermore, self-control, a core function of the individual and a key to success in life, has been associated with favorable adaptations in weight control, interpersonal relationships, academic and work performance, and decision-making (Baumeister et al., 2007; de Ridder et al., 2012; Tao et al., 2019; Wijaya and Tori, 2018). Building on H1, we further explore the role of self-control in the relationship between physical activity and career decision-making self-efficacy. Since self-control is of paramount importance in an individual's overall development and has been linked to various aspects of life success, it is reasonable to hypothesize that it may mediate the relationship. Therefore, research Hypothesis 2 is proposed (H2): self-control mediates the relationship between physical activity and career decision-making self-efficacy.

Social Anxiety is characterized as “a common human experience marked by an intense fear of evaluation from others in social situations” (Morrison and Heimberg, 2013). Relevant research has illustrated the positive effects of exercise on individuals' social anxiety (Dimech and Seiler, 2010; Deng and Wang, 2024). The potential association between social anxiety and career decision-making self-efficacy was also analyzed by integrating self-efficacy theory and labeling theory. First, from a self-efficacy theory perspective, individuals with high levels of social anxiety experience a concomitant reduction in their confidence in their social competence, leading them to feel uncertain about the social skills and competencies required in the career decision-making process, which detrimentally affects career decision-making self-efficacy (Bandura, 1977). Second, from a labeling theory perspective, when individuals are labeled by others or themselves with tags such as social anxiety disorder and social phobia, they might perceive certain jobs as unsuitable for them, which could also have a negative impact on their career decision-making self-efficacy (Bernburg, 2019). Based on the above and further expanding the exploration of factors mediating the relationship between physical activity and career decision-making self-efficacy, we propose Research Hypothesis 3 (H3): social anxiety mediates between physical activity and career decision-making self-efficacy.

Tangney et al. (2004) discovered that individuals with high self-control exhibit superior performance in interpersonal relationships, emotional responses, etc., and they concluded that poor self-control was a significant risk factor for a series of personal and interpersonal issues. Blackhart et al. (2015) also further validated through their research that poorer self-control was associated with higher social anxiety. The studies of Zhang et al. (2018) and Dong et al. (2018) found that as an individual's self-control improves, their ability to resist temptation and regulate their psychological needs is enhanced, they are more likely to be accepted and recognized by others, and their interpersonal adaptability can also be cultivated. These studies highlight the significance of self-control for individuals in interpersonal interactions. Considering these findings and the relationships established in the previous hypotheses, we propose Research Hypothesis 4 (H4): self-control and social anxiety sequentially mediate the relationship between physical activity and career decision-making self-efficacy.

### 1.1 Hypotheses of this study

**Figure 1:**
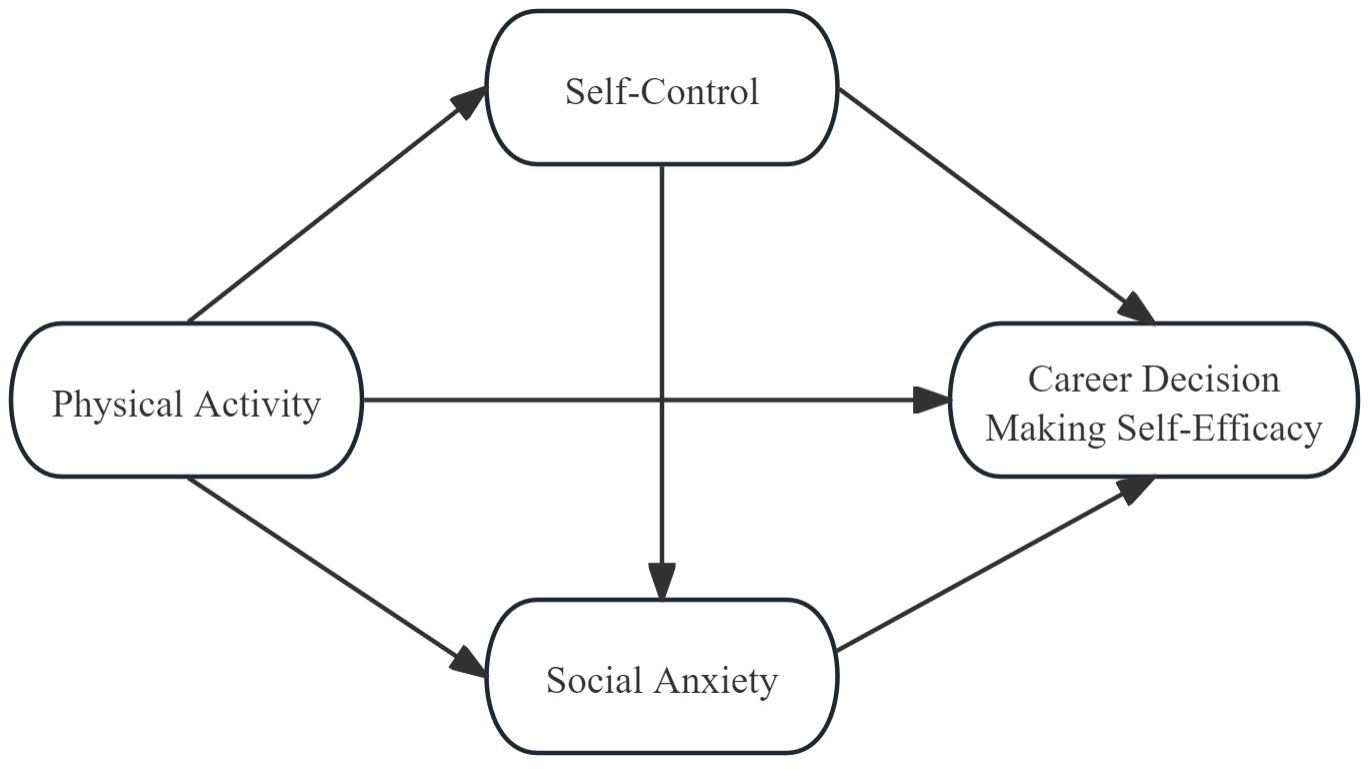
Hypothetical model.

In summary, with the aim of probing into the internal mechanism underlying the relationship between physical activity and career decision-making self-efficacy, this study constructed a chain mediation model (depicted in Figure 1) to validate the following aspects: (1) Physical activity has a significant positive predictive effect on college students' career decision-making self-efficacy. (2) Self-control mediates the relationship between physical activity and career decision-making self-efficacy. (3) Social anxiety mediates between physical activity and career decision-making self-efficacy. (4) Self-control and social anxiety sequentially mediate the relationship between physical activity and career decision-making self-efficacy. This study is devised to investigate the association between physical activity and college students' career decision-making self-efficacy and to authenticate the mediating functions of self-control and social anxiety, thereby furnishing theoretical references for augmenting college students' career decision-making self-efficacy via sports-related means.

## 2 Materials and methods

### 2.1 Measurement

#### 2.1.1 Physical activity measurement scale

The physical activity measurement scale used in this study was developed by Zou (2017). This unidimensional scale comprises five items, which are rated on a five-point Likert scale. Higher scores denote a greater degree of participation in sports activities as well as enhanced sports persistence. In this research, the overall Cronbach's α coefficient for the scale was determined to be 0.925. The results of the confirmatory factor analysis with respect to the physical activity measurement scale indicated that the model fit indices fulfilled the acceptable benchmarks. Specifically, the fit indices were detailed as follows: χ^2^/*df* = 2.624, RMSEA = 0.029, SRMR = 0.006, AGFI = 0.992, and TLI = 0.998. These data implied that the scale was possessed of satisfactory reliability and validity.

#### 2.1.2 Self-control measurement scale

The self-control scale used in this study was first developed by Tangney et al. (2004) and later modified and tested by Morean et al. (2014), Luo et al. (2021), and Fang (2022). The Chinese version of the self-control scale includes two self-discipline and impulse control constructs in a seven-item, five-point Likert scale. To ease the description of the later stage, the data were inversely processed through SPSS software, i.e., the higher the score, the better the self-control ability. In this research, the overall Cronbach's α coefficient for the scale was determined to be 0.923. The results of the confirmatory factor analysis with respect to the self-control measurement scale indicated that the model fit indices fulfilled the acceptable benchmarks. Specifically, the fit indices were detailed as follows: χ^2^/*df* = 7.399, RMSEA = 0.057, SRMR = 0.017, AGFI = 0.971, and TLI = 0.985. These data implied that the scale was possessed of satisfactory reliability and validity.

#### 2.1.3 Social anxiety measurement scale

The Social Anxiety Measurement Scale used in this study was first developed by Fenigstein et al. (1975) and later modified and refined by Scheier and Carver (1985). The Chinese version of the Social Anxiety Scale was originally developed with a 6-item, five-point Likert scale (Ma, 1999). In this study, the scale was modified appropriately through instrument checking and pilot surveys, and the scale modification process was completed by this research team after discussion, analysis, and testing. The modified scale had four items and was rated on a five-point Likert scale, with higher scores representing higher levels of social anxiety and more negative emotions and avoidance behaviors felt by individuals in social situations. In this research, the overall Cronbach's α coefficient for the scale was determined to be 0.898. The results of the confirmatory factor analysis with respect to the social anxiety measurement scale indicated that the model fit indices fulfilled the acceptable benchmarks. Specifically, the fit indices were detailed as follows: χ^2^/*df* = 3.912, RMSEA = 0.039, SRMR = 0.004, AGFI = 0.990, and TLI = 0.996. These data implied that the scale was possessed of satisfactory reliability and validity.

#### 2.1.4 Career decision-making self-efficacy measurement scale

The career decision-making self-efficacy scale used in this study was first developed by Taylor and Betz (1983) and later revised by Peng and Long (2001) and Zhao (2005). The Chinese scale version had five constructs (goal selection, self-evaluation, career information, future planning, and problem-solving) and 25 questions. It was scored on a five-point Likert scale, with higher scores indicating the higher the individual's self-evaluation or confidence in their ability to accomplish each task in the career decision-making process successfully. In this research, the overall Cronbach's α coefficient for the scale was determined to be 0.975. The results of the confirmatory factor analysis with respect to the career decision-making self-efficacy measurement scale indicated that the model fit indices fulfilled the acceptable benchmarks. Specifically, the fit indices were detailed as follows: χ^2^/*df* = 5.668, RMSEA = 0.049, SRMR = 0.022, AGFI = 0.923, and TLI = 0.968. These data implied that the scale was possessed of satisfactory reliability and validity.

### 2.2 Procedure and data processing

#### 2.2.1 Pilot survey

An instrument check was necessary to ensure that the later scales would support this study. Two professionals with doctoral degrees who worked in related research fields thoroughly checked the questionnaire's overall structure and linguistic expression. Some questions in each scale that were difficult to understand, inappropriate, or prone to ambiguity were revised and improved. For example, “Successfully coping with the job interview process” was changed to “Successfully handling the job interview process.”

The pilot survey passed the academic ethics review at the researcher's institution. The college students who participated in the survey were anonymous and volunteered, and their rights and interests will be respected and protected. The pilot survey questionnaire was distributed to college students of Qiannan Normal University for Nationalities for testing. In addition to the original questions, feedback items were set up for questions or suggestions on the questions and contents. The questionnaires were distributed to the students online. A total of 150 questionnaires were returned, among which 17 were excluded because of the identical or highly consistent answers. A total of 133 questionnaires were entered into the reliability preliminary test. The overall reliability results for each measurement in the preliminary reliability check ranged from 0.73 to 0.93. The overall reliability of social anxiety was lower than 0.8, and this study conducted further testing for the social anxiety scale. The α value of all the original items entering the reliability analysis was 0.73. The scale was processed by deleting items. The original items three and four greatly impacted the overall α value, and the two items were deleted after discussing with the research team and referencing Wu's (2010) suggestion.

#### 2.2.2 Formal survey

The formal survey utilized convenience sampling to conduct an online questionnaire survey of college students from a total of 14 colleges and universities in eight provinces of China. The formal survey passed the ethical review of the researcher's institution. The college students' participation was anonymous and voluntary, and their rights and interests were respected and protected. A total of 2,185 questionnaires were received in the formal survey. Among them, 230 questionnaires were excluded because of identical or highly consistent answers. A total of 1,955 data were included in the final data analysis, with an effective response rate of 89%. The demographic information of the participants is presented in Table 1.

**Table 1:** The demographics of the participants.

**Variables**	**Categories**	***n***	**Percentage**
Gender	Male	915	46.8%
	Female	1,040	53.2%
Major	Science and engineering	974	49.8%
	Humanities and social sciences	981	50.2%
Students' origin place	Provincial capital	101	5.2%
	Prefecture-level city	265	13.6%
	County/ County-level city	495	25.3%
	Town	325	16.6%
	Rural	769	39.3%
Parents' highest education level	Graduate (master and above)	31	1.6%
	University undergraduate/ Associate degree	229	11.7%
	High school/Vocational school	414	21.2%
	Middle school	874	44.7%
	Elementary school and below	407	20.8%

#### 2.2.3 Data analysis methods

The initial data was inputted and stored using Microsoft Excel. Subsequently, SPSS version 26.0 was utilized to conduct a comprehensive suite of statistical analyses on the data, encompassing descriptive statistics, a normal distribution test (with reference to Skewness and Kurtosis), a reliability test (with reference to Cronbach's α), an exploratory factor analysis (to address common method bias), a correlation test, and a regression analysis. In this study, in the event that the *p*-value < 0.05, the null hypothesis shall be rejected, thereby signifying that the research outcomes are statistically significant. Furthermore, the testing approach implemented is the two-tailed test. This study will construct a structural equation model via AMOS 24.0 software to validate the research hypotheses. Through the Bootstrap method, 5,000 random samplings were carried out, and the research hypotheses was examined based on whether the 95% confidence interval encompassed 0. The mediation effect was considered significant if the confidence interval did not contain 0. The specific model was configured with physical activity as the independent variable, career decision-making self-efficacy as the dependent variable, and self-control and social anxiety as the mediating variables. The fit indices and evaluation criteria of the structural equation model are as follows: χ^2^/*df* (< 3), RMSEA (< 0.08), SRMR (< 0.05), AGFI (>0.9), and TLI (>0.9). Furthermore, considering that diverse genders, majors, places of origin, and the highest education levels of parents might induce variability in the levels of physical activity, self-control, social anxiety, and career decision-making self-efficacy among college students, gender, major, the place of origin of students, and the highest education level of parents were designated as covariates during the data analysis process to ensure the precision and stability of the study's results.

## 3 Results

### 3.1 Common method bias

This study used the Harman one-factor test for common method bias (Podsakoff et al., 2003), and the results showed five factors with eigenvalues > 1. The explained rate of the first-factor variance was 25.234%, which was less than the critical value of 40% (Deng et al., 2018), so there was no serious common method bias problem in this study.

### 3.2 Correlation tests among variables

According to the descriptive statistics, the absolute value of Skewness for all variables was < 2, and the absolute value of Kurtosis was < 7. Thus, the data used in this study conformed to a normal distribution (Kim, 2013). In addition, according to Table 2, the variables physical activity, career decision-making self-efficacy, and self-control were positively and significantly associated (*r* = 0.668, *p* < 0.01; *r* = 0.404, *p* < 0.01; *r* = 0.469, *p* < 0.01). Social anxiety was significantly negatively correlated with physical activity, career decision-making self-efficacy, and self-control (*r* = −0.381, *p* < 0.01; *r* = −0.442, *p* < 0.01; *r* = −0.687, *p* < 0.01). Generally, the association between the variables in this study was significant, offering a good foundation for the subsequent regression analysis and mediation effect test.

**Table 2:** Descriptive statistics and correlation matrix among variables.

**Variables**	**M**	**SD**	**Physical activity**	**Career decision-making self-efficacy**	**Self-control**	**Social anxiety**
Physical activity	3.010	0.997	1			
Career decision-making self-efficacy	3.338	0.743	0.668^**^	1		
Self-control	3.401	0.815	0.404^**^	0.469^**^	1	
Social anxiety	2.723	0.894	−0.381^**^	−0.442^**^	−0.687^**^	1

^**^p < 0.01.

M, mean; SD, standard deviation.

### 3.3 Regression analysis

As presented in Table 3, the results of the regression analysis reveal that physical activity exerts a positive predictive effect on self-control (β = 0.429, *p* < 0.001) as well as on career decision-making self-efficacy (β = 0.546, *p* < 0.001). Meanwhile, physical activity has a negative predictive impact on social anxiety (β = −0.108, *p* < 0.001). Furthermore, self-control demonstrates a positive predictive influence on career decision-making self-efficacy (β = 0.167, *p* < 0.001). In addition, self-control also shows a negative predictive effect on social anxiety (β = −0.644, *p* < 0.001). Lastly, social anxiety exhibits a negative predictive impact on career decision-making self-efficacy (β = −0.112, *p* < 0.001).

**Table 3:** Regression analysis between the variables.

**Variables**	**Self-control**		**Social anxiety**		**Career decision-making self-efficacy**	
	**Beta (**β**)**	***t***	**Beta (**β**)**	***t***	**Beta (**β**)**	***t***
Gender	0.107	5.030^***^	0.031	1.832	0.011	0.644
Major	−0.041	−1.947	−0.022	−1.315	0.023	1.406
Students' origin place	0.060	2.692^**^	0.041	2.317^*^	−0.040	−2.323^*^
Parents' highest education level	0.050	2.254^*^	0.003	0.192	0.070	4.039^***^
Physical activity	0.429	20.269^***^	−0.108	−5.898^***^	0.546	30.064^***^
Self-control			−0.644	−36.001^***^	0.167	7.372^***^
Social anxiety					−0.112	−5.073^***^
*R* ^2^	0.179		0.488		0.510	
Δ*R*^2^	0.177		0.486		0.508	
F	85.245^***^		309.219^***^		289.582^***^	

^*^p < 0.05.

^**^p < 0.01.

^***^p < 0.001.

Gender, major, students' origin place, and parents' highest education level are covariates beta (β), standardized coefficients; *R*^2^, *R* square; Δ*R*^2^, *R* square change.

### 3.4 Mediation analysis

**Figure 2:**
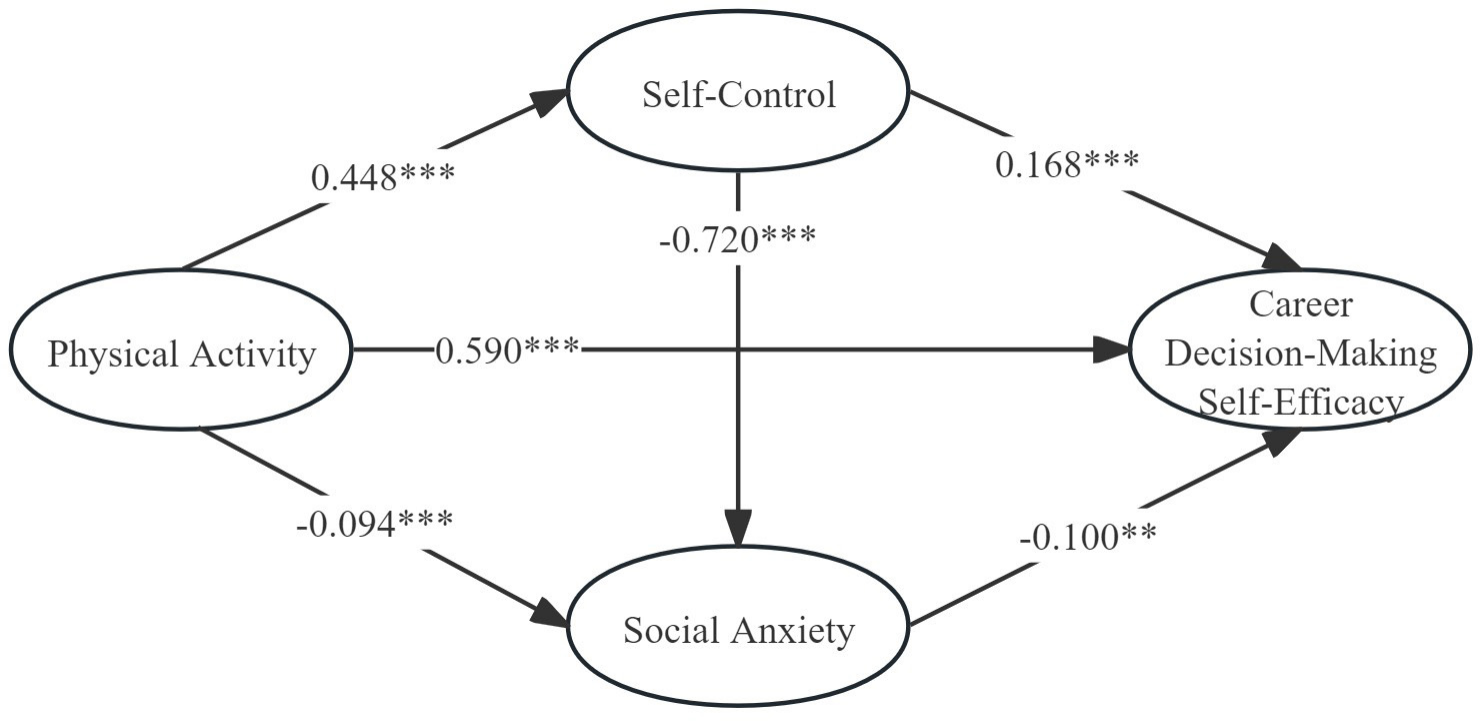
Mediation effect tests for self-control and social anxiety (normalized). ***p < 0.001, **p < 0.01.

A structural equation model was constructed using AMOS software. In this model, physical activity served as the independent variable, career decision-making self-efficacy was designated as the dependent variable, and self-control along with social anxiety functioned as the mediating variables (as depicted in Figure 2). The model fit indices were as follows: χ2/*df* = 8.236, RMSEA = 0.061, SRMR = 0.023, AGFI = 0.929, and TLI = 0.969. As presented in Table 4, the analysis results demonstrate that physical activity can significantly and positively predict career decision-making self-efficacy (Estimate = 0.590, *p* < 0.001), thereby verifying Research Hypothesis 1. As presented in Table 5, Self-control was found to exert a mediating role between physical activity and career decision-making self-efficacy, as indicated by [Estimate = 0.075, 95%CI(0.042, 0.113)]. Moreover, the magnitude of this mediating effect accounts for 10.61% of the total effect, thus verifying Research Hypothesis 2. Likewise, social anxiety was shown to play a mediating role in the relationship between physical activity and career decision-making self-efficacy, with [Estimate = 0.009, 95%CI(0.002, 0.020)]. Consequently, Research Hypothesis 3 is validated, given that the mediating effect size constitutes 1.27% of the total effect. Furthermore, a chain mediating effect was observed for the combination of self-control and social anxiety between physical activity and career decision-making self-efficacy, as demonstrated by [Estimate = 0.032, 95%CI(0.008, 0.057)]. This led to the verification of Research Hypothesis 4, where the chain mediating effect size makes up 4.53% of the total effect.

**Table 4:** Results of path analysis (normalized).

**Path**	**Estimate**	**S.E**.	**C.R**.
Physical activity **→** Self-control	0.448	0.020	18.384^***^
Physical activity **→** Social anxiety	−0.094	0.020	−4.535^***^
Self-control **→** Social anxiety	−0.720	0.028	−29.423^***^
Self-control **→** Career decision-making self-efficacy	0.168	0.028	5.337^***^
Social anxiety **→** Career decision-making self-efficacy	−0.100	0.023	−3.267^**^
Physical activity **→** Career decision-making self-efficacy	0.590	0.016	26.062^***^

^**^p < 0.01; ^***^p < 0.001.

SE, standard error; C.R., critical ratio.

**Table 5:** Mediation effect analysis (normalized).

**Path**	**Estimate**	**SE**	**Bootstrap 95% CI**	**Effect ratio**
Physical activity **→** self-control **→** career decision-making self-efficacy	0.075	0.018	(0.042, 0.113)	10.61%
Physical activity **→** social anxiety **→** career decision-making self-efficacy	0.009	0.004	(0.002, 0.020)	1.27%
Physical activity **→** self-control **→** social anxiety **→** career decision-making self-efficacy	0.032	0.012	(0.008, 0.057)	4.53%
Total mediation effect	0.117	0.013	(0.094, 0.144)	16.55%
Direct effect	0.590	0.022	(0.545, 0.631)	83.45%
Total effect	0.707	0.016	(0.674, 0.737)	100%

CI, confidence interva; SE, standard error.

## 4 Discussion

### 4.1 The relationship between physical activity and career decision-making self-efficacy

The research findings evince that physical activity is capable of significantly and positively prognosticating career decision-making self-efficacy. Hence, this intimates that physical activity has the propensity to exert a favorable influence on the career decision-making self-efficacy of college students. Primarily, physical activity exerts a favorable influence on an individual's self-efficacy (Reverdito et al., 2017). Given that career decision-making self-efficacy constitutes a component of an individual's overall self-efficacy, it will experience an increment as the overall self-efficacy escalates. More precisely, during the course of physical activities, an individual's interpersonal communication competencies, teamwork aptitudes, and stress resistance capabilities can be enhanced (Wasim et al., 2021; Décamps et al., 2012). When confronted with career decisions, individuals will appraise the congruence between their own capabilities and the corresponding occupations, as well as the amelioration in various aspects of their abilities. This, in turn, will render college students more assured when making career decisions. Secondly, through engaging in physical activities, individuals can also procure a more accurate and objective self-perception, which is of cardinal importance for aligning with target occupations and rectifying the lacunae in their extant abilities. Thirdly, considering the elemental function of sports, exercise empowers individuals to uphold sound health and cultivate a propitious body image. Exercise endows individuals with the essential health safeguard, and health functions as the bedrock for attaining career objectives. Only by maintaining a salubrious physical and mental state can individuals muster sufficient confidence to grapple with diverse challenges during the career decision-making process (Kim, 2018). Furthermore, a positive body image can augment an individual's self-assessment. Concurrently, it can also mitigate the discrimination and stereotypes that recruiters might harbor during the recruitment process. This is conducive to college students' career decision-making process (Fang, 2022; Hurley-Hanson and Giannantonio, 2006). Ultimately, upon collating all the benefits of physical activity for individuals enumerated above, on the one hand, the enhancement of these psychological traits or capabilities proffers comprehensive psychological succor to college students during the career selection process. On the other hand, these positive repercussions can be subsumed within the purview of psychological capital. In other words, physical activity can exert a beneficial influence on college students' career decision-making self-efficacy, as it expedites the accretion of individuals' psychological capital. The augmentation of psychological capital renders college students more resolute in relation to their intended occupations when making career determinations. Even when confronted with impediments and tribulations, they are convinced that they can surmount the obstacles and make unwavering choices (Gao et al., 2024; Zhou et al., 2024).

Although this study has delved into the impact of physical activity on career decision-making self-efficacy, certain limitations remain within the analysis. Future studies ought to take into account the incorporation of additional variables that are theory-related for the purpose of conducting in-depth research and analysis. Moreover, in light of the variations in the impacts exerted by diverse sports events, activity types, and their respective modalities on individuals, future studies are also required to further investigate which specific types of sports or activity modalities are more conducive to augmenting college students' career decision-making self-efficacy. Additionally, it is necessary to explore how to select appropriate sports types or activity modalities in accordance with individual differences.

### 4.2 The mediation effect of self-control

Self-control functions as a mediator between physical activity and career decision-making self-efficacy, and a positive correlation exists between physical activity and self-control. This finding aligns with the research outcomes of Boat and Cooper (2019). In relation to the nexus between physical activity and self-control, Song (1992) posited that during the acquisition of sports skills, individuals are, in essence, augmenting their control capabilities, which are predominantly exhibited in bodily movements within a three-dimensional space as well as in psychological regulation. Baumeister et al. (2006) indicated that physical activity serves to decelerate the depletion rate of individuals' “self-control resources”. In the subsequent stage of the pathway, self-control ability exerts an influence on career decision-making self-efficacy. When contemplating the constituents of self-control, such as the capacity to modify one's preponderant response and the faculty to modulate one's behaviors, thoughts, and emotions (Englert et al., 2020; Hagger et al., 2010), these regulated elements bear positive implications for the amelioration of personal competencies, social integration, academic achievements, and the proficiency in formulating and fulfilling plans. The augmentation of these capabilities plays a pivotal role in bolstering career decision-making self-efficacy, as can be discerned by integrating the sub-dimensions of the concepts of self-control and career decision-making self-efficacy. From the vantage point of self-efficacy theory (Bandura, 1977), an individual's self-assurance emanates from the perception of control. Self-control substantially augments the level of self-confidence in career decision-making by heightening an individual's sense of mastery over emotions, behaviors, and goal attainment. If an individual harbors a high degree of confidence in their self-control ability, it is evident that during the career decision-making process, when confronted with careers that appeal to them and pose challenges, they will exhibit greater confidence in their aptitude to execute tasks and surmount problems. Furthermore, career decision-making customarily entails protracted uncertainty and the capacity to defer gratification. Self-control encompasses not only the ability to regulate immediate actions but also the tenacity to adhere to long-term objectives (Mischel et al., 1989). A sound self-control ability is of cardinal importance for fostering an individual's capacity to contend with uncertainties and pressures in career selections. It should be emphasized that the enhancement of self-control ability represents not merely an amelioration of an individual's internal resources but may also indirectly impinge on career decision-making self-efficacy by influencing the individual's socialization trajectory (such as teamwork capabilities and social relationship management acumen).

In summation, college students can efficaciously enhance their self-control ability through physical activities, which facilitates the formation of positive self-assessments and the augmentation of personal capabilities. With the advancement of capabilities and the refinement of self-evaluations, college students' confidence in accomplishing diverse work tasks within the career decision-making process will likewise be fortified. For ensuing research endeavors, on the one hand, more in-depth explorations of the mediating role of self-control from the socialization perspective are warranted. On the other hand, given that the process of physical activities also drains an individual's self-control resources (Boat and Cooper, 2019), and considering the strength model of self-control (Baumeister et al., 1994), one crucial aspect that must be taken into account in future studies is that physical activities that factor in individuals' interests might yield more favorable results in this regard.

### 4.3 The mediation effect of social anxiety

Social anxiety functions as a mediator between physical activity and career decision-making self-efficacy, and a negative association exists between physical activity and social anxiety, which aligns with the findings of Dimech and Seiler (2010). With regard to the negative association between physical activity and social anxiety, first and foremost, physical activity inherently possesses its own social attributes. Through long-term engagement in physical activity, individuals can foster mutual understanding, forge social connections, and augment social support (Hikihara et al., 2018), thereby effectively enhancing an individual's social competence and alleviating social anxiety. Secondly, communication within the context of physical activity encompasses both verbal and non-verbal elements (such as eye contact, gestures, and body language). The diversity of such communication modalities may mitigate the stress induced by purely verbal exchanges. Finally, group physical activities are characterized by a shared group goal. This common objective can strengthen individual solidarity and participation, facilitate attention diversion, and reduce social anxiety by diminishing self-focus. In the latter part of the pathway, a negative association is observed between social anxiety and career decision-making self-efficacy. When college students step into the workplace, the social landscape becomes more complex, and the demands for social skills escalate. Should college students frequently experience nervousness and unease in interpersonal settings, accompanied by fearful and avoidant behaviors, this will impact their confidence level in fulfilling tasks during the career selection process, thereby constraining their career decision-making and potentially even giving rise to negative behaviors such as self-abandonment. Moreover, individuals afflicted with social anxiety are often accompanied by cognitive biases (Rheingold et al., 2003). These biases can prompt them to overstate the likelihood of failure and underrate the probability of success in career decision-making, consequently further diminishing career decision-making self-efficacy.

As a result, physical activity can alleviate their social anxiety and improve their social coping ability, which is conducive to the formation of positive self-evaluation, and with higher self-evaluation, college students' confidence in completing various work tasks in the process of career decision-making is also elevated. It should be noted that the path mediated by social anxiety has the lowest effect ratio in the path, possibly due to the light weight of social confidence in the whole career decision-making process or because of the sample collected. Caution is needed in applying this conclusion, and hopefully, future studies will be able to validate it.

### 4.4 Chain mediating effects

This study found that self-control and social anxiety sequentially mediated the relationship between physical activity and career decision-making self-efficacy. The chain mediation is achieved mainly because self-control is negatively associated with social anxiety, which is consistent with the findings of Blackhart et al. (2015). Both in terms of the definition of self-control and the evidence provided by relevant studies (Baumeister et al., 2006; Englert et al., 2020; Hagger et al., 2010), self-control encompasses control over one's behavior and emotions. First, individuals with high self-control will be more self-confident in social interaction, better able to control their emotions and behaviors during social interaction, and more likely to present a positive image in social situations, thus reducing social anxiety. Secondly, self-control ability may allow individuals to pay more attention in social situations, which, on the one hand, is beneficial to understanding others' intentions and emotions, and, on the other hand, it can also help them not to pay too much attention to their own negative emotions and others' evaluations by shifting their attention.

Therefore, college students can improve their self-control ability through physical activity. With the improvement of self-control ability, college students can better control their negative emotional and behavioral reactions, such as nervousness, fear, and avoidance in social situations, to improve their social coping ability. These changes benefit college students' positive evaluations of themselves, and with higher self-evaluations, college students are more confident in their ability to complete tasks in their career choices successfully. Moreover, it should be noted that according to the strength model of self-control (Baumeister et al., 1994), how the predictive effect of self-control on social anxiety changes when excessive psychological energy is consumed during exercise needs to be further investigated by combining the frequency, duration, and intensity of the physical activity with the different sports and types of exercise.

## 5 Limitations

Firstly, given that the present study is cross-sectional in nature, it precludes the establishment of causal relationships. It is anticipated that future research endeavors will employ a longitudinal research design to monitor the dynamic fluctuations of variables. The research participants should be selected from a diverse array of cultural backgrounds to enhance the generalizability of the findings. Secondly, certain paths within the study exhibited relatively weak effects. Hence, prudence must be exercised when interpreting and applying the results associated with these paths. Additionally, it is hoped that future investigations will conduct further validation of these paths to enhance the robustness of the findings. Finally, in consideration of the strength model of self-control and the reciprocal impacts between physical activity and self-control, forthcoming studies are obliged to integrate variables such as trait self-control, state self-control, exercise frequency, exercise duration, exercise intensity, various sport modalities, strategies for implementing physical activity, individual disparities, and exercise preferences into longitudinal experimental designs. This is of paramount importance for elucidating the triggering mechanisms and ascertaining the efficacy of augmenting college students' career decision-making self-efficacy through physical activity.

## 6 Conclusion

This study has explored the intrinsic relationship between physical activity and career decision-making self-efficacy. Physical activity significantly predicted career decision-making self-efficacy. Self-control and social anxiety played independent and chain mediating roles between physical activity and career decision-making self-efficacy. These findings have provided important theoretical support for physical activity as an effective means to enhance university students' career decision-making self-efficacy and have offered a reference for the design of sports activity programs. Furthermore, this study has provided a new perspective and direction for understanding and enhancing university students' career decision-making self-efficacy.

## Data Availability

The raw data supporting the conclusions of this article will be made available by the authors, without undue reservation.
